# A Case of Lung Cancer Unexpectedly Detected with ^99m^ Tc (V)-DMSA Whole-Body/SPECT Imaging

**DOI:** 10.1055/s-0045-1809053

**Published:** 2025-05-06

**Authors:** Esmaeil Gharepapagh, Leila Namvar, Sahar Rezaei

**Affiliations:** 1 Clinical Research Development Unit of Tabriz Valiasr Hospital, Tabriz University of Medical Sciences, Tabriz, Iran; 2 Department of Nuclear Medicine, Medical School, Tabriz University of Medical Sciences, Tabriz, Iran; 3 Tuberculosis and Lung Disease Research Center, Tabriz University of Medical Sciences, Tabriz, Iran

**Keywords:** lung cancer, ^99m^ Tc (V)-DMSA, SPECT imaging

## Abstract

In terms of cancer-related deaths, lung cancer is the most common type. To prevent this malignant and dangerous disease from progressing, early and differential diagnoses are very critical. A combination of dimercaptosuccinic acid and pentavalent ^99m^ Tc under alkaline conditions, ^99m^ Tc (V)-DMSA, can be used for malignant tumors' early diagnosis and prognosis. The ^99m^ Tc (V)-DMSA radiotracer is most commonly used for follow-up of medullary thyroid cancer metastases, but it is also successful in lung cancer and other malignancies. In this case, a 65-year-old woman with a recent history of nonproductive cough and mild dyspnea and suspicious small masses of lungs in computed tomography was selected for imaging with ^99m^ Tc (V)-DMSA to evaluate the condition of radiotracer uptake in the lung masses. The whole-body and single-photon emission computed tomography imaging with semiquantitative analysis showed abnormal uptakes in the lesions and the patient underwent to transbronchial biopsy and bronchial washing cytology that confirmed non-small cell lung cancer.

## Introduction

Lung cancer is among the most prevalent and leading causes of cancer-related deaths. 1 Public health concerns remain despite the decline in mortality in recent decades. 2 Prognosis and treatment strategies are highly affected by early diagnosis, accurate staging, response assessment, and prognostication of the localized disease. 3

For noninvasive assessment of lung cancer in a variety of clinical settings, [ ^18^ F] fluorodeoxyglucose positron emission tomography/computed tomography ([ ^18^ F] FDG-PET/CT) is widely accepted method. 4 Currently, [ ^18^ F] FDG-PET/CT is utilized for the characterization of lung lesions, staging, detecting distant metastases, and diagnosing recurrent disease, and is also a valuable tool during treatment monitoring. 5 Despite this, PET is not widely available. Furthermore, some limitations and high costs prevent [ ^18^ F] FDG-PET/CT from becoming the ideal technique.

Molecular imaging equipment available internationally is primarily gamma cameras (single-photon emission computed tomography [SPECT] modality), which represent more than 70% of the total. ^99m^ Tc is the most commonly used radionuclide for SPECT images. Thus, there is an increasing need in oncology for radiopharmaceuticals labeled with ^99m^ Tc for cancers imaging. A combination of dimercaptosuccinic acid and pentavalent ^99m^ Tc under alkaline conditions, ^99m^ Tc (V)-DMSA, can be used for malignant tumors' early diagnosis and prognosis. It is believed that ^99m^ Tc (V)-DMSA concentration in tumor tissues is related to blood volume inside tumors and phosphate metabolism. 6 This nonspecific, multifunctional imaging agent is commonly detected in medullary thyroid carcinoma, as well as head and neck, brain, lung, liver, breast, and some other soft tissues. 7 Thus, SPECT imaging of various types of cancers, including lung cancer has been improved using ^99m^ Tc (V)-DMSA. 8

## Case Report

**Fig. 1:**
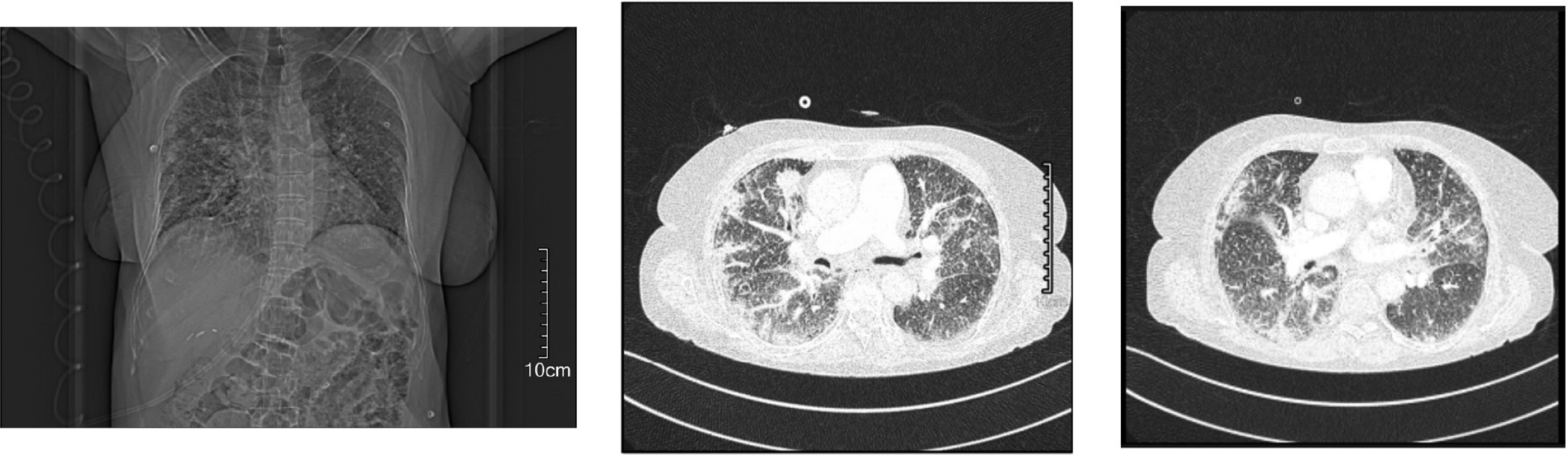
( **A** ): Plain chest X-ray illustrates some irregular opacities in both lungs without any notable masses or nodes; Chest computed tomography with contrast shows ( **B** ) mass-like patchy opacities due to lymphangitis carcinomatosis and ( **C** ) a relatively prominent focal mass in the upper lobe of the right lung in addition to patchy opacities.

A 65-year-old woman with a previous history of smoking, hypertension, and cerebrovascular accident was hospitalized for recently nonproductive cough and mild dyspnea. The plain chest X-ray showed some irregular opacities in both lungs without remarkable mass or nodes ( Fig. 1A ). For control, she underwent chest CT scan with contrast that showed pulmonary opacities as well as a focal mass in both lungs randomly identified in the report ( Fig. 1B and C ). The prominent mass had a diameter of 32 mm in the upper lobe of the right lung. The probable diagnosis was carcinomatosis lymphangitis due to the right upper lung lesion and interstitial edema, therefore, further evaluation was recommended.

**Fig. 2:**
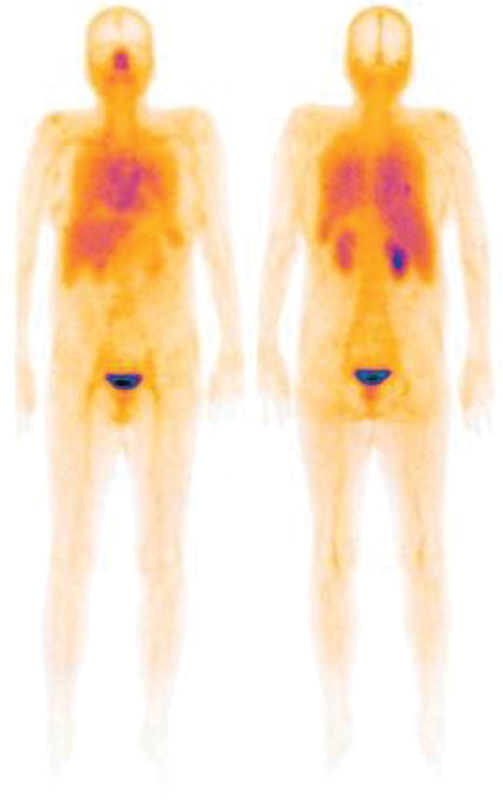
Scan demonstrates two lesions with increased radiotracer uptake in right and left lungs.

**Fig. 3:**
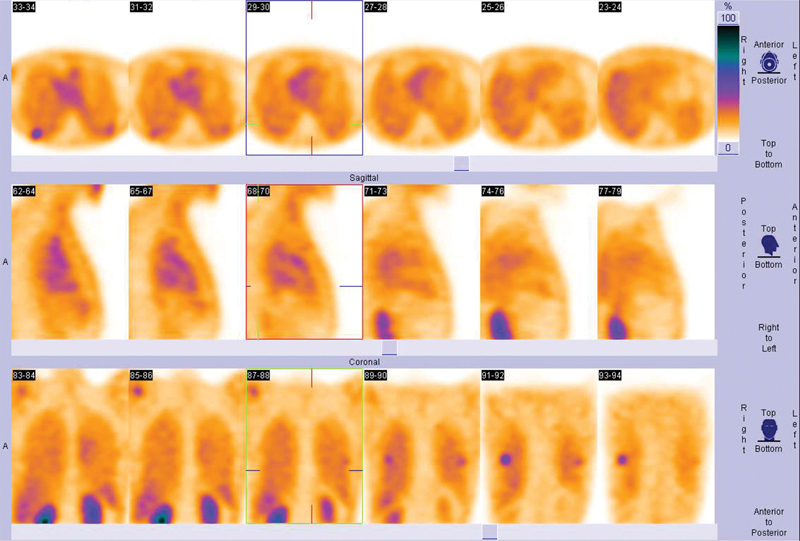
Single-photon emission computed tomography projections illustrate lung lesions posteriorly on both sides.

The control CT scan with contrast performed 7 days later and revealed the presence of same masses without changes, also several enlarged lymph nodes in the mediastinum and lungs' hilum, and no evidence of thrombosis or pulmonary artery stenosis on both sides. For further evaluation of pulmonary lesions ^99m^ Tc (V)-DMSA imaging was requested by the attending physician. The whole-body scan as well as thoracic SPECT images were taken 2 hours after the intravenous injection of 590 MBq of radiotracer using a dual head Siemens gamma camera with low energy high resolution (LEHR) collimators. The scan was interpreted by nuclear medicine specialist and showed two lesions with increased radiotracer uptake, one in the lower region of right upper lobe and the other in the superior segment of left lung ( Fig. 2 ). The lesions had posterior locations in both lungs best detected by SPECT projections ( Fig. 3 ). In semiquantitative analysis, the lesion to background ratio (L/B) was 1.60 on the right side and 1.50 on the left side. Also, there were some patchy lesions with slightly increased uptake in the upper and middle parts of both lungs. The possibility of malignancy in two obvious lesions in the right and left lungs was issued based on the ^99m^ Tc (V)-DMSA scan report. As a result, lung transbronchial biopsy and bronchial washing cytology were performed. It is imperative to note that most lung lesions were sampled and biopsied. Nonsmall cell lung cancer was identified in the results. The chemotherapy was started for this patient.

## Discussion

It has long been known that CT presents limitations when it comes to detecting metastatic nodules and separating viable residual tumors from fibrotic tissue; this has led to several alternative approaches being investigated. 9 PET is currently recognized as a modality for the workup and ongoing monitoring of a wide range of malignant tumors, particularly lung cancer. 10 In spite of this, PET has some limitations, such as not being widely available and being very expensive. In this regard, whole-body imaging with ^99m^ Tc (V)-DMSA is cheap, and noninvasive.

In patients with thyroid medullary carcinoma, ^99m^ Tc (V)-DMSA scanning is an imaging method widely accepted and used. 11 Additionally, ^99m^ Tc (V)-DMSA has been shown to uptake breast cancer, 12 lung cancer, 13 head and neck cancer, 14 pituitary adenomas, 15 bone metastases, and other bone lesions. 16 It is known that ^99m^ Tc (V)-DMSA accumulates in tumor cells due to its pH-sensitive property. 17 Tumors that are malignant or aggressive tend to have an acidic pH, produce lactic acid at a high rate, and promote hexokinase and glucose transporter overexpression. 18 Accordingly, the mechanism of ^99m^ Tc (V)-DMSA uptake for malignant tumors appears to be similar to that of FDG, since FDG accumulates primarily due to increased glucose utilization in cancer cells, increased activity of hexokinase, and glucose transporters. 19 20 21 Therefore, ^99m^ Tc (V)-DMSA is capable of detecting malignant lung lesions and their metastases as well as monitoring the response to chemotherapy and radiotherapy in patients who are unable to undergo surgery.

Images obtained with planar ^99m^ Tc (V)-DMSA are only sufficient to detect peripheral lesions and are not able to clearly display deeper lesions within the lungs. ^99m^ Tc (V)-DMSA scans are limited by their low sensitivity to detect blood pool malignancies, such as in the mediastinum. 6 Thus, SPECT imaging is critical and gives valuable insight into deep-seated lesions using ^99m^ Tc (V)-DMSA scan. 7 Besides its ideal characteristics as a radiolabel, ^99m^ Tc (V)-DMSA is easy to prepare on site, making it a relatively inexpensive modality for metabolic imaging. For optimal results, a single and late ^99m^ Tc (V)-DMSA SPECT scan is adequate. As we know, tissue sampling is necessary to make a precise diagnosis in this and all other cases suspected of malignancy. However, physicians can make more informed decisions regarding further lung mass evaluation with early imaging with ^99m^ Tc (V)-DMSA, which is a quick, simple, and inexpensive method.
